# Single-Trial Evoked Potential Estimating Based on Sparse Coding under Impulsive Noise Environment

**DOI:** 10.1155/2018/9672871

**Published:** 2018-03-22

**Authors:** Nannan Yu, Ying Chen, Lingling Wu, Hanbing Lu

**Affiliations:** ^1^School of Electrical Engineering and Automation, Jiangsu Normal University, Xuzhou 221116, China; ^2^Department of Internal Neurology, Xuzhou Central Hospital, Xuzhou 221116, China

## Abstract

Estimating single-trial evoked potentials (EPs) corrupted by the spontaneous electroencephalogram (EEG) can be regarded as signal denoising problem. Sparse coding has significant success in signal denoising and EPs have been proven to have strong sparsity over an appropriate dictionary. In sparse coding, the noise generally is considered to be a Gaussian random process. However, some studies have shown that the background noise in EPs may present an impulsive characteristic which is far from Gaussian but suitable to be modeled by the *α*-stable distribution (1 < α ≤ 2). Consequently, the performances of general sparse coding will degrade or even fail. In view of this, we present a new sparse coding algorithm using *p*-norm optimization in single-trial EPs estimating. The algorithm can track the underlying EPs corrupted by *α*-stable distribution noise, trial-by-trial, without the need to estimate the *α* value. Simulations and experiments on human visual evoked potentials and event-related potentials are carried out to examine the performance of the proposed approach. Experimental results show that the proposed method is effective in estimating single-trial EPs under impulsive noise environment.

## 1. Introduction

Evoked potentials (EPs) are time-locked biological signals recorded from the scalp in response to a variety of well-defined external stimuli [1]. Depending on the modality of stimulation, EPs are categorized into auditory (AEPs), visual (VEPs), somatosensory (SEPs), and motor (MEPs) evoked potentials. EPs contain several components that can be distinguished according to their respective latencies and amplitudes [2]. The latency variations of specific components can objectively reflect changes in the underlying state of the neural pathways, which is very meaningful in cognitive science research and clinical applications, such as brain-computer interface, the diagnosis of possible brain injury, and the intraoperative monitoring [3, 4]. Many single-trial EP extracting methods have been proposed in order to enhance the ability to track latency variations [5].

EP signals have time-locked (quasi-periodic) characteristics and are always accompanied by nonstationary ongoing electroencephalogram (EEG) signals. Moreover, the signal-to-noise ratio (SNR) of EP records is usually low (0 to −30 dB). Estimating single-trial EPs corrupted by EEG can be regarded as signal denoising problem. Sparse coding is a powerful tool for the analysis of nonstationary signals [6, 7]; it has achieved significant success in signal denoising and separation. Huang et al. [8] proposed the mixed overcomplete dictionary-based sparse component decomposition method (MOSCA), which decomposes the EP and EEG signals in the wavelet dictionary (WA) and discrete cosine transform (DCT) dictionary, respectively. However, the WA and DCT dictionaries cannot meet completely the characteristics of EPs and EEG. Their partial components are represented by the wrong dictionaries and their corresponding coefficients. Therefore, MOSCA cannot separate the EP and EEG signals sufficiently. To solve this problem, we proposed a dictionary construction method for the EP signal and a double-trial estimation method based on joint sparse representation [9].

Traditionally, for mathematical convenience, the noise in EP signals is considered to be a Gaussian random process. However, some studies have shown that the background noise in clinical EP signals is often impulsive non-Gaussian distributed [10]. Consequently, the EP estimation algorithms developed under a Gaussian background noise assumption may fail or be not optimal. That is, the impulsive feature in the noise may cause the performance of algorithms based on the second-order moment (SOM) to degrade or even fail. The *α*-stable distribution is a widely used class of statistical distributions for impulsive non-Gaussian random processes [11]. In comparison with a Gaussian process, an *α*-stable process often has many more sharp spikes in its realization and a probability density function (PDF) with a heavy tail [12, 13]. It has been shown that an *α*-stable (1 < *α* ≤ 2) process is more suitable for modeling the background noise in EP observations than is a Gaussian process because the noise is often impulsive and its PDF has a heavy tail. This will degrade the performance of the sparse coding algorithm.

In this paper, we present a novel approach to solving the EP estimating problem under impulsive noise environment based on sparse coding using least mean *p*-norm (SC-LMP) optimization. It has been proven that least mean *p*-norm algorithm always works if *p* is set to 1 when 1 < *α* ≤ 2 [14]. So in SC-LMP, in order to facilitate solving the sparse coefficients, the 1-norm is used in place of the *p*-norm. We then formulate the minimization of the cost function into a linear programming (LP) problem. The EPs can be reconstructed by the sparse coefficients and the dictionary. Experimental results show that the SC-LMP algorithm can work well when the *α* value dynamically changes. It can track latency variations even in situations of extremely low SNR. The rest of this paper is organized as follows.  Section 2 gives a detailed description of our single-trial estimation algorithm.  Section 3 contains our experimental results obtained by using the SC-LMP method and a comparison with traditional sparse coding methods with least-mean-square (LMS) optimization and MOSCA.  Section 4 presents our conclusions.

## 2. Single-Trial Evoked Potential Estimation with SC-LMP

Numerous studies have shown that in EPs the background noise is found to be non-Gaussian and suitable to be modeled by the *α*-stable distribution. The main parts of our method consist of removing the noise *e*(*t*) from the measurement *y*(*t*) and then reconstructing the single-trial EP *s*(*t*). The measurement *y*(*t*) is

(1) $$\begin{array}{c} y\left(\;t\;\right)=s\left(\;t\;\right)+e\left(\;t\;\right), \end{array}$$

where *s*(*t*) is a time-locked signal and *e*(*t*) is a zero-mean *α*-stable distribution process. A fractional lower-order *α*-stable (FLOA) distribution is obtained if 0 < *α* < 2 for an *α*-stable distribution. One distinct feature of an FLOA process is that there are more samples far away from the mean or the median than those of a Gaussian process. Thus, the wave forms of FLOA observations have many more impulsive spikes.

### 2.1. *1-Norm* Cost Function

Estimating single-trial evoked potentials (EPs) corrupted by the spontaneous electroencephalogram (EEG) can be regarded as signal denoising problem. A least square (2-norm) approach is commonly used. However, it has been shown that the background noise in EPs may present an impulsive characteristic which is far from Gaussian but suitable to be modeled by the *α*-stable distribution (0 < *α* < 2). Compared with *L*_2_*-*norm, *L*_*P*_*-*norm is a better option.

Sparse coding is a powerful tool in analysing nonstationary signals, and it has shown significant success in signal denoising and separation. And in our previous papers [9], we have proved that EPs have strong sparsity over an appropriate dictionary. The EPs can be represented as

(2) $$\begin{array}{c} s\left(\;t\;\right)=D\theta , \end{array}$$

where *D* ∈ *R*^*M*×*N*^ is the dictionary and *θ* ∈ *R*^*N*×1^ is the sparse coefficient.

The EP estimating problem can be solved using sparse coding with least mean *p*-norm (SC-LMP) optimization. The cost function is

(3) $$\begin{array}{c} E\left(\;\theta \;\right)={\left\|\;y\left(\;t\;\right)-D\theta \;\right\|}_{p}+\lambda {\left\|\;\theta \;\right\|}_{1}. \end{array}$$

It has been proven that the least mean *p*-norm algorithm always works if *p* is set to 1 when 1 < *α* < 2. So in SC-LMP, in order to facilitate solving the sparse coefficients, the *L*_1_-norm is used in place of the *p*-norm. So the function can be rewritten as

(4) $$\begin{array}{c} E\left(\;\theta \;\right)={\left\|\;y\left(\;t\;\right)-D\theta \;\right\|}_{1}+\lambda {\left\|\;\theta \;\right\|}_{1}. \end{array}$$

The problem for the estimation of *θ* by minimizing (4) could be formulated into

(5) minθ Pθ−Y1where P=DλIN×N,  Y=yt0N×1,

where 0 denotes the vector of all zeros with appropriate size.

### 2.2. Optimization

In order to solve the optimization problem in (5), we formulate the problem as a LP problem as follows. Let *x* = *Pθ* − *Y*, *x*^+^ = max⁡(*x*, 0), and *x*^−^ = max⁡(−*x*, 0). Then *x* can be expressed as *x*^+^ − *x*^−^. The minimization problem can now be rewritten as

(6) minθ,x+,x− 1Tx++1Tx−s.t. Pθ−Y=x+−x− x+,x−≥0,

where 1 denotes the vector of all ones with appropriate size. The equation above can be written as a LP problem in a standard form as follows:

(7) minx qTxs.t. Ax=Ywhere q=011,  x=θx+x−,  A=P−II.

Then we can solve the LP problem using linear interior point solver (LIPSOL), which is based on a primal-dual interior point method.

(8) q=011,x=θx+x−,A=P−II.

### 2.3. Reconstructing

After solving (7), we can use the solution *x* to reconstruct the single-trial EP $\hat{s}\left(t\right)$ as follows:

(9) $$\begin{array}{c} \hat{s}\left(\;t\;\right)=D\hat{\theta }. \end{array}$$

## 3. Experiment Results

**Figure 1:**
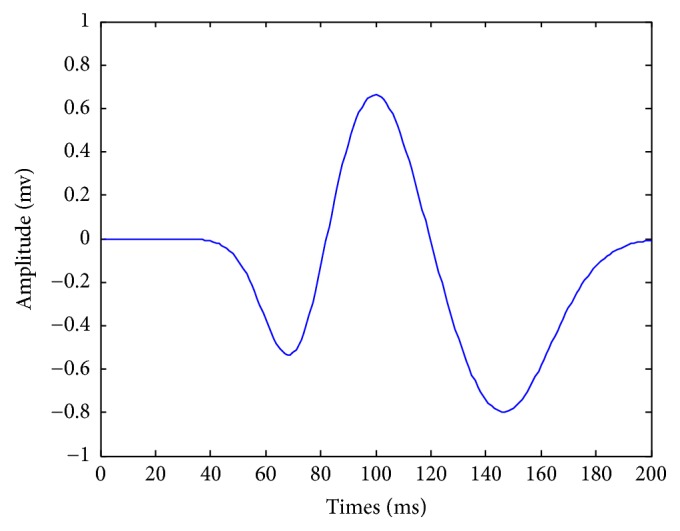
Stimulated EP.

Computer simulation was conducted to verify the performance of the SC-LMP algorithm for EP signal estimation under FLOA noise environments. The simulated EP data is constructed by superimposing three Gauss distribution functions [15] and the waveform is shown in Figure 1; thus,

(10) st,m=−0.6exp⁡−t−75−m2152+0.7exp⁡−t−100+m2202−0.8exp⁡−t−145−m2252.

FLOA noise with various *α* values was generated to simulate background noise. The observations were additive mixtures of the noise-free signals and the simulated FLOA background noise. The mixed signal-to-noise ratio (MSNR) is defined as follows:

(11) $$\begin{array}{c} MSNR=10\mathrm{lg}\left(\;\frac{\sigma_{s}^{2}}{\gamma_{v}}\;\right), \end{array}$$

where *σ*_*s*_^2^ and *γ*_*v*_ are the variance of the noise-free signal and the dispersion of the FLOA background noise, respectively. Two estimation algorithms, namely, LMS-RBFNN [16] and ARX [17], were compared in the following simulations. In ARX, the *s*(*t*, 0) is used as the exogenous input to the estimated ARMA (autoregressive-moving-average) model; the model order is estimated by FPE [18] and the parameters are calculated by LMS [19]. To measure the performance of the algorithms, the correlation coefficient *ρ* is defined as

(12) $$\begin{array}{c} \rho =\frac{\sum_{t=0}^{M-1}\left(s\left(t,m\right)-\overset{-}{s}\right)\left(\hat{s}\left(t,m\right)-\overset{-}{\hat{s}}\right)}{\sqrt{\sum_{t=0}^{M-1}{\left(s\left(t,m\right)-\overset{-}{s}\right)}^{2}}\sqrt{\sum_{t=0}^{M-1}{\left(\hat{s}\left(t,m\right)-\overset{-}{\hat{s}}\right)}^{2}}}, \end{array}$$

where $\overset{-}{s}$ and $\overset{-}{\hat{s}}$ are the time mean values with *M* samples of *s*(*t*, *m*) and $\hat{s}(t,m)$.

### 3.1. Simulation Experiment

**Figure 2:**
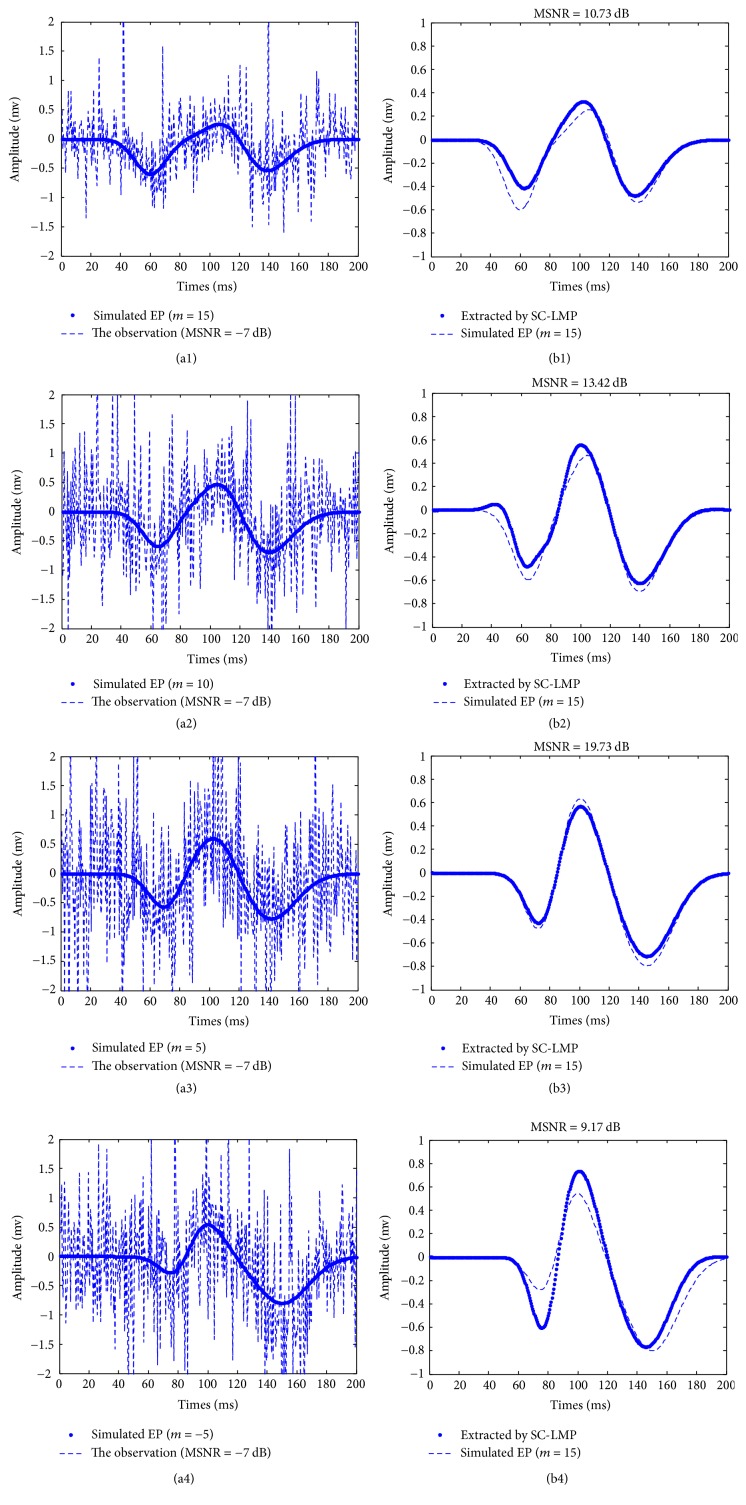
Single-trial EPs *s*(*t*, *m* = 15,10,5, −5) with MSNR = −7 dB estimated using our method.

In this section, the proposed method is compared with two other methods, namely, ARX and LMS-RBFNN. ARX and LMS-RBFNN are one of the commonly used methods to extract EP signal. ARX modeling for single-trial EP estimation was proposed by Cerutti et al. [20]. This method can estimate single-trial EPs even when the SNR is very low and has been applied to the monitoring of the depth of anesthesia during surgery. RBENN is a kind of supervised feedforward neural network based on function approximation theory. Fung et al. [21] proposed LMS-RBFNN method according to the strong approximation ability and fast training speed of RBENN.  Figure 2 shows 4 graphs of the estimated single-trial EP signals based on our method. Figures 2(a1)–2(a4) include stimulated EP in various latencies (*m* = 15,10,5, −5) which are indicated by dotted line and the accordingly observed signals are mixed by MSNR = −7 dB which are indicated by dashed-dotted line. Figures 2(a1)–2(a4) show the accordingly estimated results by SC-LMP. From Figure 2, we can see that, with the increase of the value of the MSNR, our method has better dynamic estimation ability of latency and amplitude in different *m* value.

**Figure 3:**
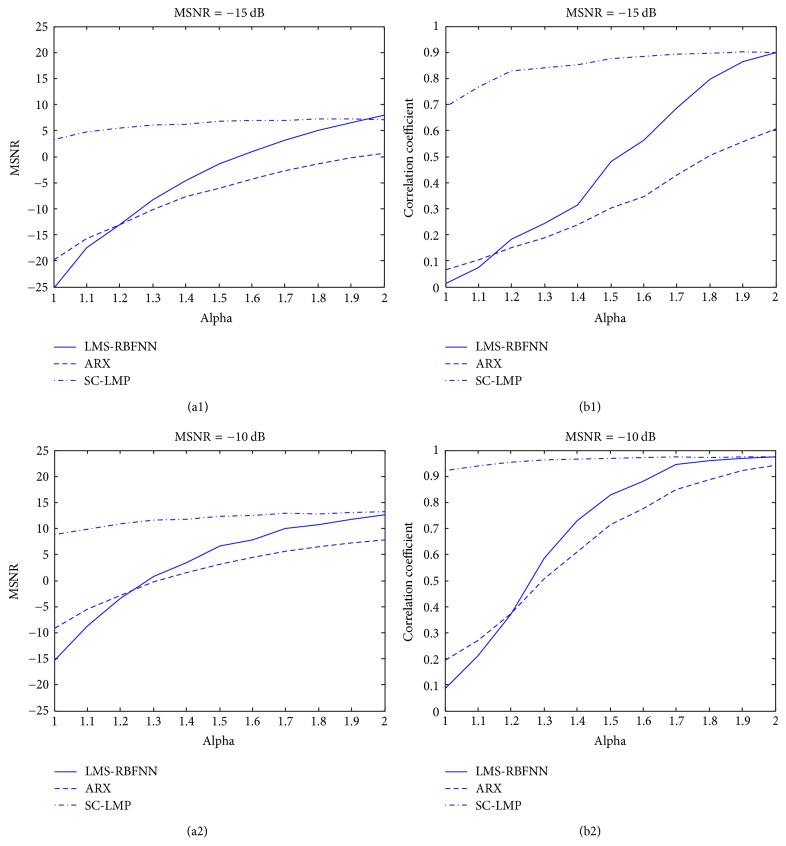
Comparison of three methods in different alpha values.

As shown in Figure 3, we changed *α* value from 1 to 2 and calculated the improvement of MSNR and the correlation coefficient in the corresponding MSNR value (MSNR = −15, −10 dB) obtained with our method, ARX and LMS-RBFNN. Compared with ARX and LMS-RBFNN, our method exhibits better performance, with slight decreasing of MSNR when alpha varies from 2 to 1.

**Figure 4:**
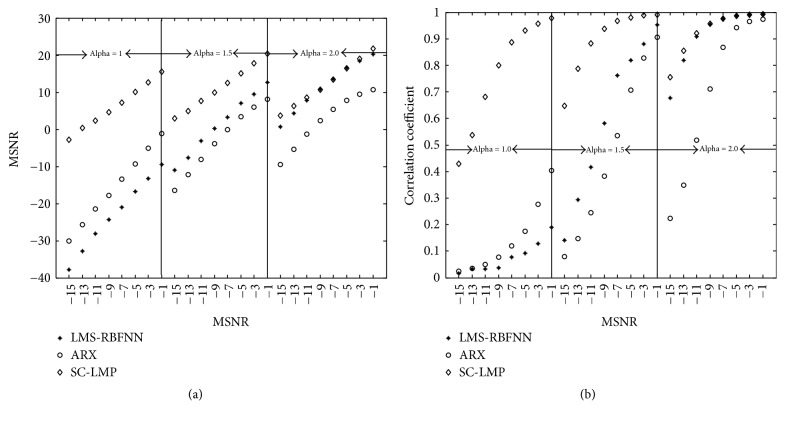
Comparison of three methods in different MSNR values.

The improvement of MSNR and the correlation coefficient of our method, ARX and LMS-RBFNN in three alpha values (alpha = 1, 1.5, and 2), are shown in Figure 4. From Figure 4, with the decrease of the value of MSNR, the estimated value of MSNR and the correlation coefficient of three methods decline. However, compared with the other 2 methods, our method has better performance.

### 3.2. Real Data

For further evaluation of the performance of our method, real VEPs were used by [22]. We chose a small piece of data for trial. The data was then rereferenced to the average of channels O1, Oz, and O2, low-pass filtered between 0 and 9 Hz with a 7th-order Butterworth filter, and downsampled to 128 Hz.

**Figure 5:**
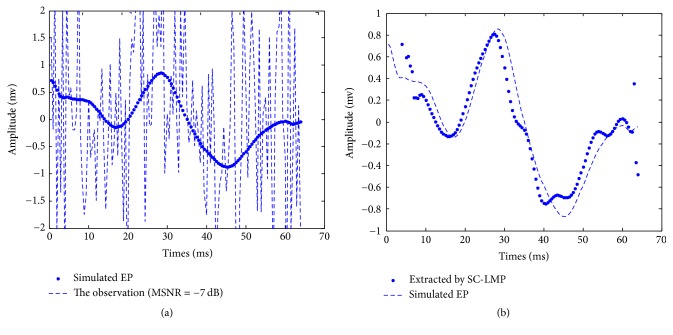
The extracted result by using real data.

Figure 5(a) shows the stimulated EP and the accordingly observed signals which are the mixture of the stimulated EP and *α*-stable distribution noise by MSNR = −7 dB. We extract the EP with SC-LMP, and results are shown in Figure 5(b). Clearly, the signal estimated using our method better resembles the stimulated EP. The component P300 of VEPs extracts with our method is distinct.

## 4. Conclusion

To sum up, we proposed a novel single-trial EP estimated method based on SC-LMP. This method uses sparse coding to represent EPs and utilize a zero-mean *α*-stable distribution process to express spontaneous EEG according to the characteristics of background signal. In order to facilitate solving the sparse coefficients, the *p*-norm is used in place of the *L*2-norm. We conducted a series of experiments on simulated and real data, and the results were evaluated using waveform extractions and other metrics. As the experimental results show, our method has better estimated capacity and performance than other existing algorithms. Future works will focus on improving the stability and practicability of the new proposed method to obtain a better real-time monitoring of the components. This could lead to the development of more advanced applications for real-world signals.
